# Validation of control genes and a standardised protocol for quantifying gene expression in the livers of *C57BL/6* and *ApoE*−*/*− mice

**DOI:** 10.1038/s41598-018-26431-3

**Published:** 2018-05-24

**Authors:** Priscilla E. L. Day, Karen F. Chambers, Mark S. Winterbone, Tatiana García-Blanco, David Vauzour, Paul A. Kroon

**Affiliations:** 1 Food & Health Programme, Quadram Institute Bioscience, Norwich Research Park, Norwich, Norfolk NR4 7UA UK; 2 Norwich Medical School, University of East Anglia, Norwich Research Park, Norwich, Norfolk NR4 7UQ UK

## Abstract

The liver plays a critical role in food and drug metabolism and detoxification and accordingly influences systemic body homeostasis in health and disease. While the *C57BL/6* and *ApoE*−*/*− mouse models are widely used to study gene expression changes in liver disease and metabolism, currently there are no validated stably expressed endogenous genes in these models, neither is it known how gene expression varies within and across liver lobes. Here we show regional variations in the expression of *Ywhaz*, *Gak*, *Gapdh*, *Hmbs* and *Act-β* endogenous genes across a liver lobe; Using homogeneous samples from the four liver lobes of 6 *C57BL/6* mice we tested the stability of 12 endogenous genes and show that *Act-β* and *Eif2-α* are the most stably expressed endogenous genes in all four lobes and demonstrate lobular differences in the expression of *Abca1* cholesterol efflux gene. These results suggest that sampling from a specified homogeneous powdered liver lobe is paramount in enhancing data reliability and reproducibility. The stability of the 12 endogenous genes was further tested using homogeneous samples of left liver lobes from 20 *ApoE*−*/*− mice on standard or high polyphenol diets. *Act-β* and *Ywhaz* are suitable endogenous genes for gene expression normalisation in this mouse model.

## Introduction

The *C57BL/6* mouse model is a widely known inbred mouse strain that has been used in numerous studies to investigate disease phenotypes and outcomes in response to treatments such as in alcoholic liver diseases and dietary treatments respectively^1–4^. Due to its ability to evoke spontaneous hypercholesteraemia and arterial lesions resembling those of human atherosclerosis as well as its propensity to be induced to a full spectrum non-alcoholic steatohepatitis (NASH), an ApoE knock-out (*ApoE*−*/*−*)* mouse model is widely used for studying the underlying mechanisms involved in cardiometabolic disease development^5–7^. Food bioactive compounds such as polyphenols have been shown to be protective against cardiometabolic diseases through anti-lipidaemia and anti-inflammatory mechanisms^5,8^. Although the liver has several lobes with reported vascular anatomical and physiological variations which may contribute to molecular differences, numerous studies have utilised livers from both *C57BL/6* and *ApoE*−*/*− models without necessarily reporting how samples were collected^9–11^. Understanding whether such molecular variations exist between and within lobes with respect to gene expression is paramount for data reliability as well as for improving data reproducibility and consequently reducing the number of animals being used in such studies in accordance with the “3 Rs” (Replacement, Reduction and Refinement) in animal ethics^12^. Therefore, we determined whether there are differences in gene expression in different biopsies taken from the same liver lobe or between different liver lobes. Polyphenols are known to induce gene expression changes in the liver. However, most studies investigating the underlying mechanisms involved in the protective effects of polyphenols have used single, endogenous genes such as 18S ribosomal RNA (*18S*), glyceraldehyde-3-phosphate dehydrogenase (*Gapdh*) and beta-actin (*Act-β*) for gene expression normalisation without the validation of their expression stability^13,14^. It is widely accepted that single genes may not be suitable for gene expression normalisation as certain endogenous genes including *Gapdh* and *Act-β* may be regulated under certain pathological conditions or treatment and may change depending on experimental conditions and cell type^15^. *18S* ribosomal RNA, has also been shown to be a poor representation of mRNA expression^15^. As such, the use of these genes without the validation of their stability may severely compromise assay sensitivity and accuracy leading to inaccurate data interpretation. In the current study, we sought to validate stably expressed endogenous genes in the liver lobes of *C57BL/6* fed a standard diet and *ApoE*−*/*− mice fed a low polyphenol diet or tomatoes containing different types but similar amounts of polyphenols (Table 1).

**Table 1:** Diets fed to the *ApoE*−*/*− mice and their phenolic components.

Diet	Main polyphenols
Standard	Low polyphenol red tomato
Flavonol	Kaempferol
Flavonol + Anthocyanins	Kaempferol + Delphinidin
Resveratrol	Resveratrol
Isoflavones	Genestin

## Results

**Figure 1:**
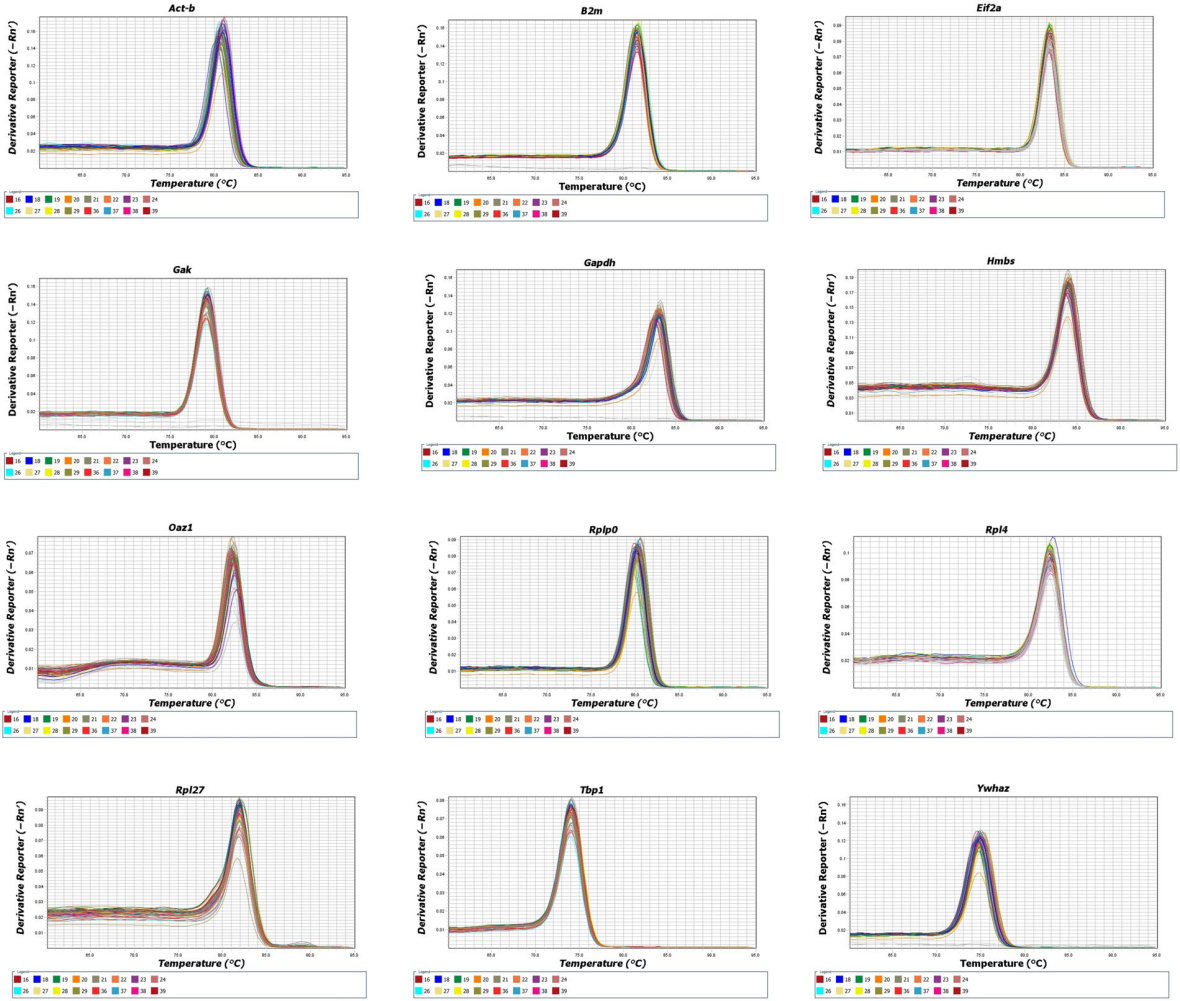
Melt curve values of the endogenous genes measured in this study.

While mRNAs from twelve endogenous genes were quantified in this study, *Rpl4* and *Oaz1* were eliminated from the Bestkeeper analysis due to their amplification efficiencies being lower than 90% and the software having a limit of 10 genes per analysis. As such the stabilities of ten endogenous genes; *Ywhaz*, *Act-β*, *Tbp*, *Gak*, *Rpl27*, *Hmbs*, *Rplp0*, *Gapdh*, *β2m and Eif2-*α were determined using the Bestkeeper software, while the stabilities of all the genes were analysed using the Normfinder software. These genes had amplification efficiencies ranging from 90 to 112% and their regression coefficient was between 0.96 and 0.99 (Table 2). The melt curve for all the genes also showed a single PCR amplification peak indicating that the primers were specific for the target genes investigated (Fig. 1).

**Table 2:** Primer sequences and PCR efficiencies and information about genes used in this study.

Gene	Full gene name	Gene ID	Product size	Forward primer	Reverse primer	R^2^ Value	Amplification efficiency
*Ywhaz*	Tyrosine 3-monooxygenase/tryptophan 5-monooxygenase activation protein, zeta polypeptide	NM_011740.3	78	CCAGACTGAGGAAGATTAAGCAAT	CAGTTCCAGGTATCATTTGTAATTT	0.97	108
*Act-β*	βeta-actin	NM_007393.5	104	CTAAGGCCAACCGTGAAAAG	ACCAGAGGCATACAGGGACA	0.99	98
*Tbp1*	TATA box binding protein	NM_013684	187	ATCAACATCTCAGCAACC CA	TTG AAG CTG CGGTACAAT TC	0.99	108
*Gak*	Cyclin G associated kinase	NM_153569.1	111	GGTCATCCAGTCTGTGGCTAAC	TTGATTGCAGACTCCACACC	0.99	112
*Rpl27*	Ribosomal protein L27	NM_000988.4	80	TGAAAGGTTAGCGGAAGTGC	TTTCATGAACTTGCCCATCTC	0.96	90
*Hmbs*	Hydroxymethylbilane	NM_013551.2	250	GAATTCAGTGCCATCGTCCT	CTTCTGGGTGCAAAATCTGG	0.99	103
*Rplp0*	60 S acidic ribosomal protein P0	NM_007475.5	124	ACTGGTCTAGGACCCGAGAAG	CTCCCACCTTGTCTCCAGTC	0.98	100
*Gapdh*	Glyceraldehyde-3-phosphate dehydrogenase	NM_001289726.1	266	ACAGTCCATGCCATCACTGCC	GCCTGCTTCACCACCTTC TTG	0.99	105
*β2m*	β−2-microglobulin	NM_009735.3	241	CTGCTACGTAACACAGTTCCACCC	CATGATGCTTGATCACATGTCTCG	0.99	94
*Eif2-α*	Eukaryotic translation elongation factor 1 alpha 2	NM_026114.3	78	ACTTTTAGTAAGGATGGGACATTGTT	TCCCTTGTTAGCGACATTGA	0.97	92
*Rpl4*	Ribosomal protein L4	NM_024212.4	95	AGCAGCCGGGTAGAGAGG	ATGACTCTCCCTTTTCGGAGT	0.99	82
*Oaz1*	Ornithine Decarboxylase Antizyme 1	NM_001301034.1	121	CTCTGCCTGAGGGCAGTAAG	AGTAGGGCGGCTCTGTCC	0.83	65

**Figure 2:**
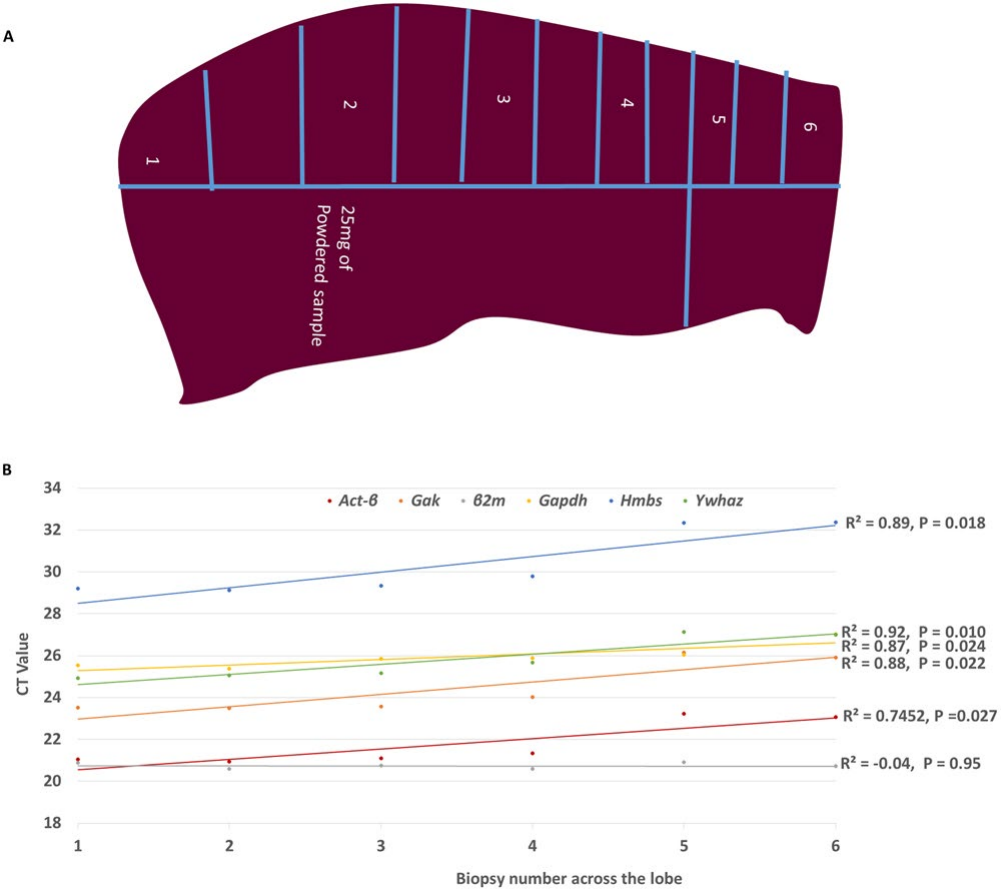
Biopsy sampling in mice lobes and gene Ct value correlation with position of sampling. The Ct values for the housekeeping genes *Ywhaz*, *Gak*, *Gapdh*, *Hmbs* and *Act-β* were positively correlated to sampling positions along the vertical section of the left lobe but there was no correlation between the Ct value of *β2M* and sampling positions along the vertical section.

Initially the expression of six putative housekeeping genes was used to determine whether there are any variations in expression between biopsies obtained from a single liver lobe. The expression of five endogenous genes was positively correlated to the biopsy location along the liver lobe, with *β2m* being the only endogenous gene that did not differ across the liver lobe (Fig. 2).

**Figure 3:**
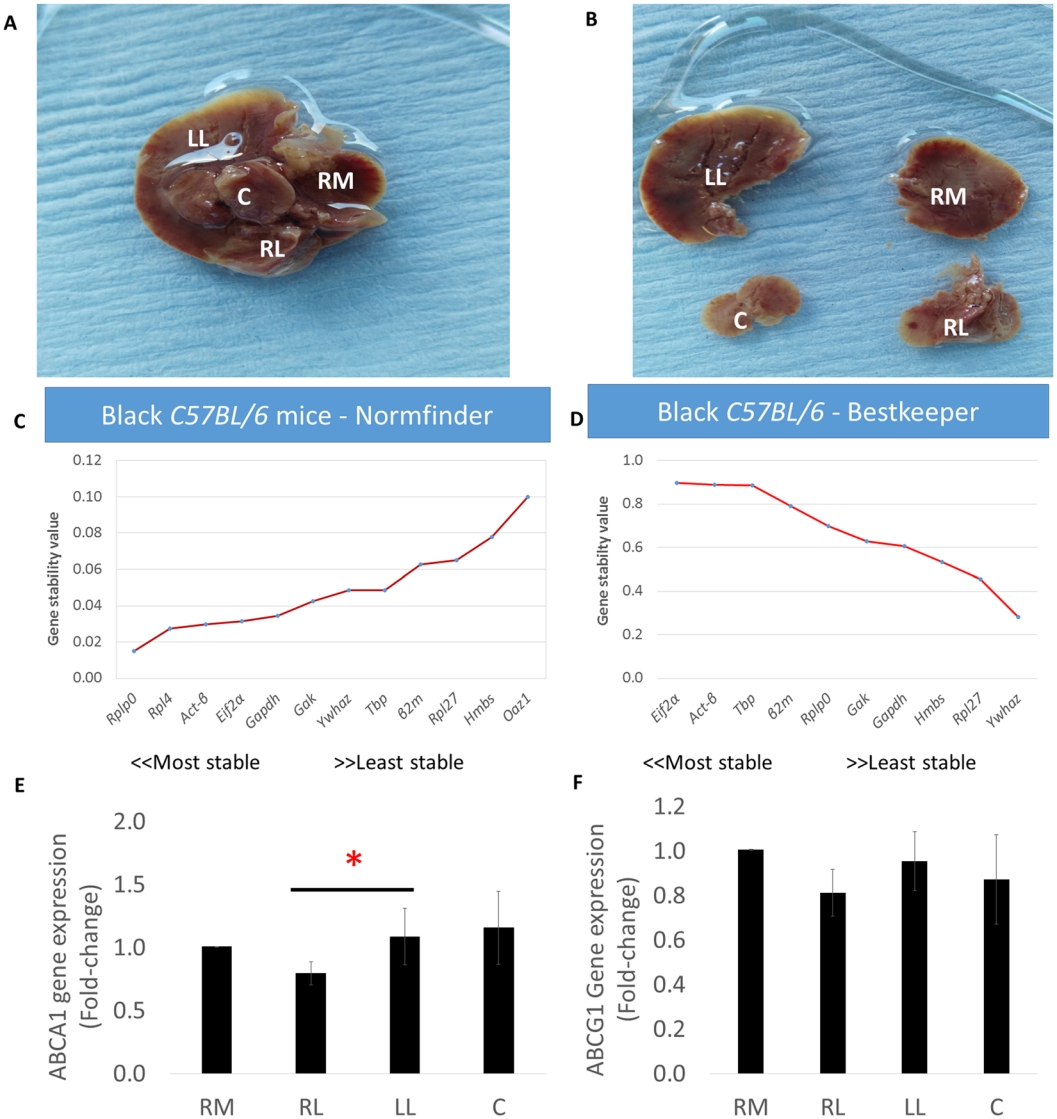
The four liver lobes, gene expression stability results and the expression of cholesterol efflux genes. (**A**,**B**) Lobular liver sampling, (**C**,**D**) Ranked gene stability by Normfinder and BestKeeper algorithms in the different liver lobes and (**E**,**F**) Lobular differences in the expression *Abca1* and *Abcg1* genes. RM (right medial lobe), RL (Right lateral lobe), LL (left lobe), C (Caudate lobe). *Abca1* and Abcg1data were normalised to RM values for each mouse and data are mean ± SEM *p ≤ 0.05 (Gene expression normalised to the geometric mean of *Act-β* and *Eif2-α*).

We then investigated the expression of the endogenous genes in the four liver lobes (Fig. 3A,B). The Normfinder software identified *Rplp0*, *Rpl4*, *Act-β* and *Eif2-α* as the most stably expressed endogenous genes in the different liver lobes in this order, while *Rpl27*, *Hmbs* and *Oaz1* were the least stably expressed endogenous genes (Fig. 3C). *Rplp0*, *Act-β* and *Eif2-α* remained the most stably expressed genes even *when Rpl4* and *Oaz1* were eliminated from the Normfinder analysis. The results were similar to those generated by the Bestkeeper software in that *Eif2-α* and *Act-β* were the most stably expressed endogenous genes, although *Rplp0* ranked 5^th^ rather than as the most stably expressed gene. Although the expression stability of *Tbp* as shown by Bestkeeper was also closer to that of *Act-β* and it was ranked as the third most stably expressed gene in the different liver lobes, it was ranked 8^th^ by Normfinder (Fig. 3D). Next, we determined the expression of two genes involved in cholesterol efflux namely; *Abca1* and *Abcg1* in the four lobes. We thus used the geometric mean of *Act-β* and *Eif2-α* to normalise the gene expression of the cholesterol efflux genes *Abca1* and *Abcg1* in the different mouse lobes. The expression of *Abca1* was significantly (P = 0.028) lower in the right lateral lobe than in the left lobe and it tended to be higher in the right lobe compared to the right medial lobe and the caudate lobe, although this did not reach significance. (Fig. 3E). While there were no significant differences in the expression of *Abcg1* between the lobes, its expression tended to be lower in the right lateral lobe compared to the other lobes (Fig. 3F).

**Figure 4:**
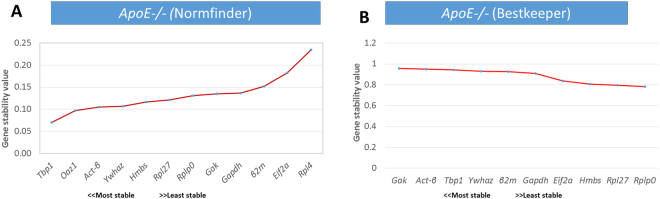
Gene stability in the *ApoE*−*/*− mouse livers fed a purified standard or different types of polyphenol diets. (**A**) Ranked gene stability by Normfinder and (**B**) ranked by BestKeeper algorithms.

The Normfinder analysis which included all twelve genes indicated that *Tbp*, *Oaz1*, *Act-β* and *Ywhaz* are the most stably expressed genes in this order in the *ApoE*−*/*− mouse livers fed on a standard low polyphenol diet and different types of high polyphenol diets (Fig. 4A). Although *Oaz1* was eliminated from the Bestkeeper stability analysis, the algorithm also indicated that *Gak*, *Act-β*, *Tbp* and *Ywhaz* are the most stably expressed endogenous genes (Fig. 4B).

Overall, the results from the two algorithms are complimentary in that *Act-β*, *Tbp* and *Ywhaz* are the most stably expressed genes even when *Oaz1* and *Rpl4* were eliminated from the Normfinder analysis. However, Bestkeeper indicated that *Gak* was the most stably expressed endogenous gene but Normfinder ranked *Gak* among the least stably expressed genes. These discrepancies can also be seen with *Hmbs* and *Rpl27* which were among the least stably expressed according to Bestkeeper and they may be explained by the differences in which the two algorithms calculate the most stably expressed gene^16–19^.

**Figure 5:**
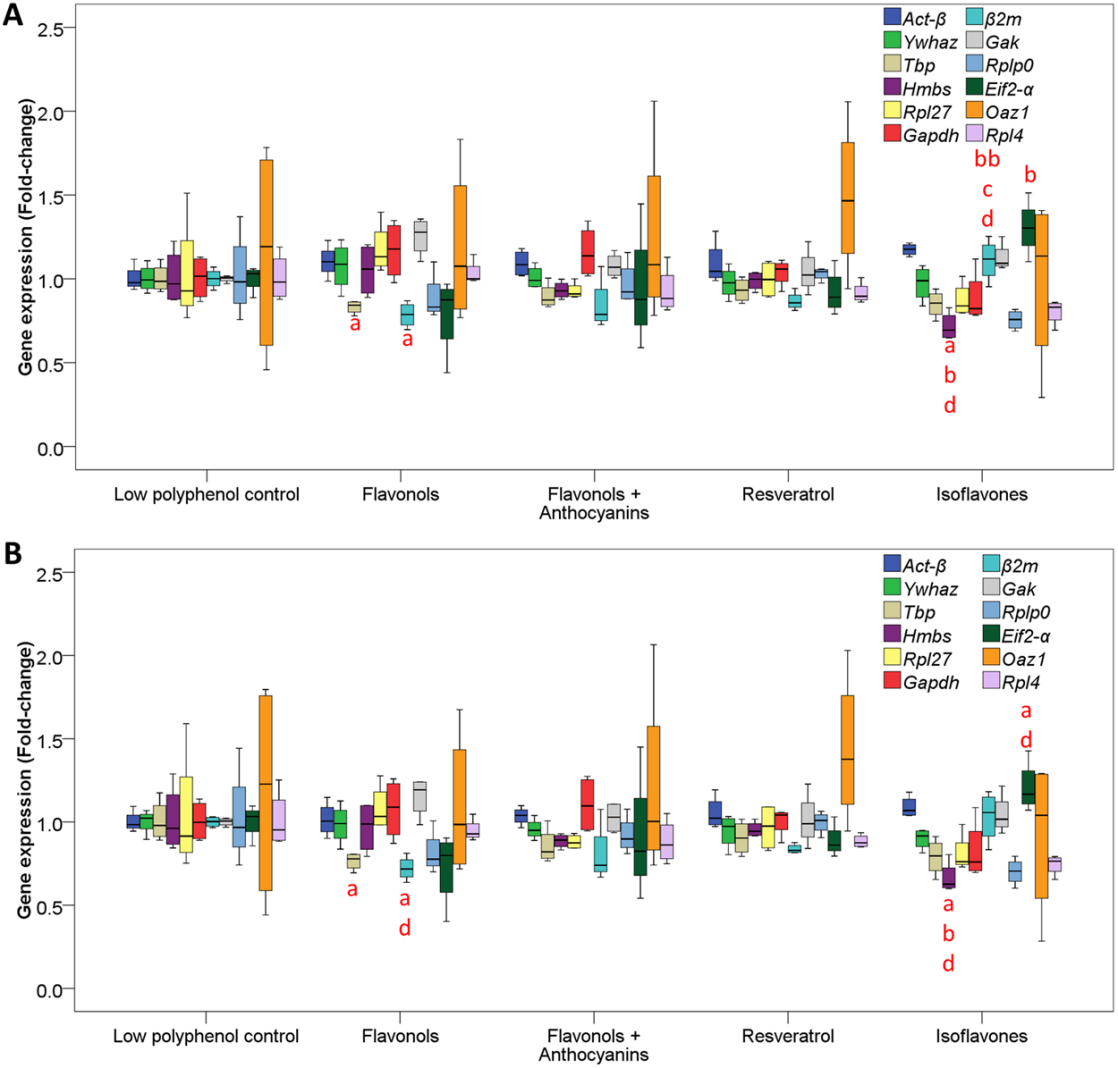
Box and whisker plots of gene expression differences between polyphenol treated groups *of ApoE*−*/*− mice. (**A**) Gene expression normalised to *Act-β*, *Tbp and Ywhaz*. (**B**) Gene expression normalised to *Act-β*, *and Ywhaz* (single letter symbols mean P = 0.05 and double letter symbols mean P ≤ 0.01, ^a^difference from control, ^b^difference from flavonol, ^c^difference from flavonol + anthocyanins and ^d^difference from resveratrol). Data presented as median, upper and lower values and interquartile ranges.

Next, we assessed the expression of the twelve genes between the different groups using the geometric mean of *Tbp*, *Act-β* and *Ywhaz* as control genes. Despite *Tbp* being one of the most stably expressed genes, its expression together with that of *Gak*, *Hmbs*, *β2M* and *Eif2-α* was significantly different between the groups (Fig. 5A). We then assessed the expression of the twelve genes after eliminating *Tbp* as one of the control genes (i.e, only using *Act-β* and *Ywhaz* as endogenous genes) and this also indicated that together with *Hmbs*, *β2m*, and *Rpl4*, the expression of *Tbp* was significantly different between the five groups. However, the expression of *Gak* was no longer different between the groups (Fig. 5B).

## Discussion

Sampling methodologies, size and number can influence data reproducibility, particularly in tissues such as the liver which has been shown to have both anatomical and physiological lobular differences. Coupled with the use of endogenous genes that may not be stably expressed depending on treatment and disease phenotype for gene expression normalisation, this can lead to erroneous data interpretation. In this study, we initially compared the Ct values of endogenous genes in the biopsies of a left liver lobe which showed a positive correlation of sampling from the apex of the lobe to the end which joins the portal and arterial vascularization and the biliary drainage. Indeed, several other studies have reported variations in the vascular systems to and from the lobes both in humans and animals^10,20^. Whereas the use of sample biopsies from human livers cannot be avoided, data from such studies should be interpreted with caution. However, in animal studies where it is possible to obtain whole liver lobes the use of liver biopsies should be avoided as this may lead to data misrepresentation. Several studies have reported changes in the expression of hepatic genes in response to treatments or disease phenotypes without necessarily reporting on sampling methodologies^21–23^. Here we show that there are differences in the expression of *Abca1* cholesterol efflux gene in the different liver lobes and although not statistically significant, we show a trend towards a reduction in *Abcg1* expression in the right lateral lobe in comparison to other lobes. This suggests that comparing samples collected from different lobes could potentially introduce data interpretation biases. To our knowledge, this is the first time that lobular differences in gene expression have been reported in livers from mice. However, these results are in line with gene expression differences observed in different lobes of mid-gestation fetal baboons and in physiological and biochemical studies that have shown lobular variations in hepatic toxicity and fibrotic changes after paracetamol and carbon tetrachloride treatments respectively^11,24,25^. Furthermore, differences in the distribution of minerals such as iron, copper and phosphorus have also been observed in addition to differences in tracer uptake which have been attributed to the variations in blood flow between the liver lobes^26^. Thus, we recommend that future studies only compare data obtained from a homogeneous powder of a specified liver lobe, preferably the left lobe as this provides a much bigger sample that could be used for other analyses such as the quantification of metabolites and thus allow the comparison between gene expression and cellular metabolism.

While the algorithms for determining stably expressed endogenous genes indicated that in *ApoE*−*/*− mice livers fed on a standard low polyphenol diet or those on a high polyphenol diets *Tbp*, *Act-β* and *Ywhaz* are the most stably expressed genes, it was surprising to find that there are differences in the expression of *Tbp* between groups. The reason for this is not clear but it suggests that *Tbp* cannot be reliably be used as a control gene in an *ApoE*−*/*− mouse model and *ApoE*−*/*− mouse model on high polyphenol diets. It also highlights the need to carefully assess putative stably expressed genes within and between groups. It is also important to note that although the Normfinder algorithm takes into account inter- and intra-group differences, it still suggested that *Tbp* is one of the most stably expressed genes. With these observations in mind, we recommend that further analyses should be considered in order to determine whether the so called stably expressed endogenous genes are not differentially expressed between groups before they are used for gene expression normalisation.

Although *Gapdh* has been used in several studies using an *ApoE*−*/*− mouse model to normalise gene expression, here we show that that *Gapdh* is among the least stably expressed endogenous genes across different liver lobes together with *Eif2-α*^27,28^. Instead, we recommend that *Act-β* and *Ywhaz* are suitable endogenous genes for gene expression normalisation when using *ApoE*−*/*− mice livers from mice fed on a standard low polyphenol diet or those on high polyphenol diets. For studies using *C57BL/6* mouse model where comparisons between liver lobes are required, *Act-β* and *Eif2-α* would be ideal for gene expression normalisation.

The main findings of this study are (1) that there are intra-lobular and inter-lobular differences in the expression of genes in the mouse liver, (2) Housekeeping genes *Act-β* and *Eif2-α* would be ideal for gene expression normalisation where comparisons between liver lobes in the *C57BL/6* mouse model are required and (3) that *Act-β* and *Ywhaz* are suitable endogenous genes for gene expression normalisation when using *ApoE*−*/*− mice livers from mice fed on a standard low polyphenol diet or those on high polyphenol diets. These findings have important implications in that using liver sample biopsies may lead to erroneous results and this may also occur where samples from different lobes are being compared in different treatments. We therefore suggest that a homogeneous sample from a specified liver lobe should be used in all studies where comparisons between treatments or disease phenotypes are required. In order to facilitate data comparisons between laboratories we recommend that where possible the left liver lobe should be used for gene expression studies as this provides a larger sample that can allow gene expression correlations with other molecular and biochemical markers. Alternatively, a homogeneous sample from the whole liver should be used and this should be specified in the literature.

## Methods

### Animal and sample processing

All experimental procedures and protocols used in this study were reviewed and approved by the Animal Welfare and Ethical Review Body (AWERB) at the University of East Anglia and were conducted within the provisions of the Animals (Scientific Procedures) Act 1986 (ASPA) and the LASA Guiding Principles for Preparing for and Undertaking Aseptic Surgery (2010). Care of the animals for the duration of the study was in accordance with the UK Home Office guidelines. Briefly male *C57BL/*6 were bred inhouse and 8-week-old male homozygous B6.129P2-Apoe^tm1Unc/J^ (named *ApoE*−*/*− hereafter) mice were purchased from Jackson Laboratories (Charles River Laboratories, Kent, and United Kingdom). Mice were housed in a temperature-controlled environment (22 °C) with a 12 h light/dark cycle. They were kept under relative humidity of 55% and supplied with free access to water and food with the location of each cage within the experimental room being regularly moved. C57BL/6 were fed a standard breeding diet AIN-93G until aged 8 weeks when they were sacrificed (Testdiets, Kent, United Kingdom). *ApoE*−*/*− mice were fed a standard breeding diet AIN-93G (Testdiets, Kent, United Kingdom) for 8 weeks before being randomly divided into five groups and fed ad libitum for seventeen weeks either supplemented with a low polyphenol tomato powder, or high polyphenol tomato powders where the tomatoes were enriched in either flavonols, a mixture of flavonols and anthocyanins, resveratrol or isoflavones (Table 1). The body weight and food consumption of the mice were measured three times a week and mice were sacrificed by exsanguination under isoflurane anaesthesia and perfused with 0.9% Saline and EDTA via cardiac puncture. Livers were then immediately collected and stored in RNA*later* (Life Technologies, Paisley, UK) at 4 °C overnight and kept at -20 °C until the day of RNA extraction.

### RNA extraction and CDNA synthesis

For lobular differences, 4 liver lobes were collected from six *C57BL/6* mice (Fig. 1), for within lobe variations 25 mg biopsies were obtained from different sections of the left liver lobe (Fig. 2) and for the rest of the *ApoE*−*/*− mice, the left lobe was collected. All samples were immediately transferred into RNAlater kept at 4 °C overnight and stored at −20 °C until the day of processing when they were processed into a homogeneous powder using a pestle and mortar under liquid nitrogen. 700 µl QIAzol Lysis Reagent (Qiagen, UK) was added to 25 mg of all samples and homogenised using the Precellys 24 lysis & homogeniser at 6000 rpm for 4 cycles for 30 s (Bertin Technologies, France) and total RNA was extracted using miRNeasy Mini Kits according to the manufacturer’s instructions (Qiagen, UK). Total RNA concentration was assessed by Nanodrop and 1 µg of total RNA was used for cDNA synthesis using the Precision NanoScript Reverse Transcription kit (Primerdesign, UK) in a final volume of 20 µl according to the manufacturer’s instructions.

### Real-time quantitative PCR

12 pairs of intron-spanning primers were designed using either the Roche probe library software (https://lifescience.roche.com/en_gb/brands/universal-probe-library.html) or the PubMed primer designing tool (https://www.ncbi.nlm.nih.gov/tools/primer-blast/) (Table 2) and checked for specificity using BLAST (https://blast.ncbi.nlm.nih.gov/Blast.cgi) or sent for sequencing (Eurofins, UK) (Table 2 and Supplementary Figs 1 and 2). Primers were synthesised by Integrated DNA Technologies (IDT, Belgium).

Gene expression analysis was carried out using the VIIA™ 7 PCR System (Life Technologies, UK) in a final reaction volume of 10 µl, and comprised of 1 X ImmoMix PCR MasterMix (Bioline, UK), SYBR Green (0.06ul of 100x stock), ROX reference dye (175 nM) magnesium (0.5 mM), BSA (50 µg/ml) and 10 nM forward and reverse primers. The following PCR cycling conditions were used; initial denaturation at 95 °C for another 10 min, followed by amplification and data acquisition at 95 °C for 15 sec and annealing/extension at 60 °C for 1 min for 40 cycles and a melt curve. For each gene, the melt curves (Fig. 1) and standard curves were performed to determine the primer specifity and linearity respectively (Table 2). Standard curves were also used to calculate amplification efficiencies (Table 2). All samples were run in triplicate with a no template control for each gene.

### Data analysis and statistics

We considered three programmes; Bestkeeper, Normfinder and GeNorm that apply statistical algorithms to determine the most stably expressed endogenous genes in different cell types, disease phenotypes and treatments^15,16,18^. Several studies have shown that the results obtained from these packages are fairly similar^29^. Owing to the former two packages being freely available to be used as excel add-ins, in this study Bestkeeper and Normfinder were used for endogenous gene stability analysis. Bestkeeper computes a value termed Bestkeeper index based on the geometric mean of the cycle threshold (Ct) values of each candidate gene and carries out a pair-wise correlation and regression analysis to generate P values, with the most stably expressed gene having a P value closer to 1. Normfinder determines gene stability based on inter-and intra-group variations with the most stably expressed gene having the lowest stability value.

After PCR analysis by VIIA7, data were exported to MS Excel. For analysis by Bestkeeper, raw Ct values were used to calculate the geometric mean of Ct values for all the genes to generate the Bestkeeper index, which was then correlated to the Ct values of each gene to derive the coefficient of correlation value [r]. For Normfinder a standard curve was used to calculate transcript concentrations and these were log transformed by the software and the data were used to compute stability values based on intra-group and inter-group variations. Statistics were carried out using IBM SPSS software (IBM SPSS Statistics for Windows, version 22.0; IBM Corp., Armonk, NY). For comparisons between lobes a non-parametric Wilcoxon related sample test was used. For comparisons between treatment groups, all data except *Rpl27* in the isoflavone and *Gapdh* in the resveratrol groups were normally distributed according to the Shapiro-Wilk test and as such a One-Way ANOVA with Bonferroni correction was used. *Rpl27* AND *Gapdh* data were log transformed and they remained not normally distributed and as such group differences were determined by the Kruskal-Wallis test.

### Data availability statement

The datasets generated during and/or analysed during the current study are available from the corresponding author on reasonable request.

## Electronic supplementary material

Supplementary Figure 1

Supplementary Figure 2

## References

[1] Bavia L, de Castro IA, Isaac L (2015). C57BL/6 and A/J Mice Have Different Inflammatory Response and Liver Lipid Profile in Experimental Alcoholic Liver Disease. Mediators Inflamm.

[2] Li L (2017). Characteristics of IL-9 induced by Schistosoma japonicum infection in C57BL/6 mouse liver. Sci Rep.

[3] Gangadaran S, Cheema SK (2017). A high fat diet enriched with sea cucumber gut powder provides cardio-protective and anti-obesity effects in C57BL/6 mice. Food Res Int.

[4] Wang D (2017). Green tea infusion protects against alcoholic liver injury by attenuating inflammation and regulating the PI3K/Akt/eNOS pathway in C57BL/6 mice. Food & function.

[5] Feng M (2017). Comparative effect of berberine and its derivative 8-cetylberberine on attenuating atherosclerosis in ApoE−*/*− mice. Int Immunopharmacol.

[6] Matziouridou C, Marungruang N, Nguyen TD, Nyman M, Fak F (2016). Lingonberries reduce atherosclerosis in Apoe(−*/*−) mice in association with altered gut microbiota composition and improved lipid profile. Molecular nutrition & food research.

[7] Schierwagen R (2015). Seven weeks of Western diet in apolipoprotein-E-deficient mice induce metabolic syndrome and non-alcoholic steatohepatitis with liver fibrosis. Sci Rep.

[8] Xu ZR (2015). Apple Polyphenols Decrease Atherosclerosis and Hepatic Steatosis in ApoE−*/*− Mice through the ROS/MAPK/NF-kappaB Pathway. Nutrients.

[9] Aller MA (2012). A half century (1961–2011) of applying microsurgery to experimental liver research. World J Hepatol.

[10] Malarkey DE, Johnson K, Ryan L, Boorman G, Maronpot RR (2005). New insights into functional aspects of liver morphology. Toxicol Pathol.

[11] Heinloth AN (2004). Gene expression profiling of rat livers reveals indicators of potential adverse effects. Toxicol Sci.

[12] National Centre for the Replacement, Refinement and Reduction of Animals in Research (NC3Rs), https://www.nc3rs.org.uk/the-3rs, Retrieved, 27th April, 2018.

[13] Ripley BJ (2010). Green tea polyphenol epigallocatechin gallate inhibits cell signaling by inducing SOCS1 gene expression. Int Immunol.

[14] Kessoku T (2016). Resveratrol ameliorates fibrosis and inflammation in a mouse model of nonalcoholic steatohepatitis. Sci Rep.

[15] Vandesompele J (2002). Accurate normalization of real-time quantitative RT-PCR data by geometric averaging of multiple internal control genes. Genome Biol.

[16] Andersen CL, Jensen JL, Orntoft TF (2004). Normalization of real-time quantitative reverse transcription-PCR data: A model-based variance estimation approach to identify genes suited for normalization, applied to bladder and colon cancer data sets. Cancer Research.

[17] Martinez-Beamonte R (2011). Selection of reference genes for gene expression studies in rats. J. Biotechnol..

[18] Pfaffl MW, Tichopad A, Prgomet C, Neuvians TP (2004). Determination of stable housekeeping genes, differentially regulated target genes and sample integrity: BestKeeper–Excel-based tool using pair-wise correlations. Biotechnol Lett.

[19] Svingen, T., Letting, H., Hadrup, N., Hass, U. & Vinggaard, A. M. Selection of reference genes for quantitative RT-PCR (RT-qPCR) analysis of rat tissues under physiological and toxicological conditions. *Peerj* **3**, 10.7717/peerj.855 (2015). 10.7717/peerj.855 PMC4375968 25825680

[20] Martins PNA, Neuhaus P (2007). Surgical anatomy of the liver, hepatic vasculature and bile ducts in the rat. Liver Int..

[21] Heyman-Linden L (2016). Berry intake changes hepatic gene expression and DNA methylation patterns associated with high-fat diet. Journal of Nutritional Biochemistry.

[22] Heidker RM, Caiozzi GC, Ricketts ML (2016). Grape Seed Procyanidins and Cholestyramine Differentially Alter Bile Acid and Cholesterol Homeostatic Gene Expression in Mouse Intestine and Liver. PloS one.

[23] Yonamine, C. Y. *et al*. Resveratrol Improves Glycemic Control in Type 2 Diabetic Obese Mice by Regulating Glucose Transporter Expression in Skeletal Muscle and Liver. *Molecules* (*Basel*, *Switzerland*) **22**, 10.3390/molecules22071180 (2017). 10.3390/molecules22071180 PMC6152102 28708105

[24] Keigo Y (2004). Lobular Difference in Fibrotic Changes in Rat Cirrhosis Model Induced by Carbon Tetrachloride. Toxicologic Pathology.

[25] Cox LA (2006). Gene expression profile differences in left and right liver lobes from mid-gestation fetal baboons: a cautionary tale. J Physiol.

[26] Jacobsson H, Jonas E, Hellstrom PM, Larsson SA (2005). Different concentrations of various radiopharmaceuticals in the two main liver lobes: a preliminary study in clinical patients. J Gastroenterol.

[27] Ngalame NN, Micciche AF, Feil ME, States JC (2013). Delayed temporal increase of hepatic Hsp70 in ApoE knockout mice after prenatal arsenic exposure. Toxicol Sci.

[28] Zhong CY (2015). Microbiota prevents cholesterol loss from the body by regulating host gene expression in mice. Scientific reports.

[29] De Spiegelaere, W. *et al*. Reference Gene Validation for RT-qPCR, a Note on Different Available Software Packages. *PloS one* **10**, 10.1371/journal.pone.0122515 (2015). 10.1371/journal.pone.0122515 PMC4380439 25825906

