# Supplementary material for: Validation of control genes and a standardised protocol for quantifying gene expression in the livers of C57BL/6 and ApoE−/− mice
Source: Sci Rep. 2018 May 24;8:8081. doi: 10.1038/s41598-018-26431-3 (PMC5967315; doi:10.1038/s41598-018-26431-3)
Supplement: Supplementary file 1 — Supplementary Figure 1 [file 41598_2018_26431_MOESM1_ESM.pdf]

Sequencing product and chromatogram from forward primer (CTA AGG CCA ACC GTG AAA AG) - GACCTTCAACACCCCAGCCATGTACGTAGCCATCCAGGCTGTGCTGTCCCTGTATGCCTCTGGT

Note: Positions 3, 8 were called manually as a C and an A, respectively.

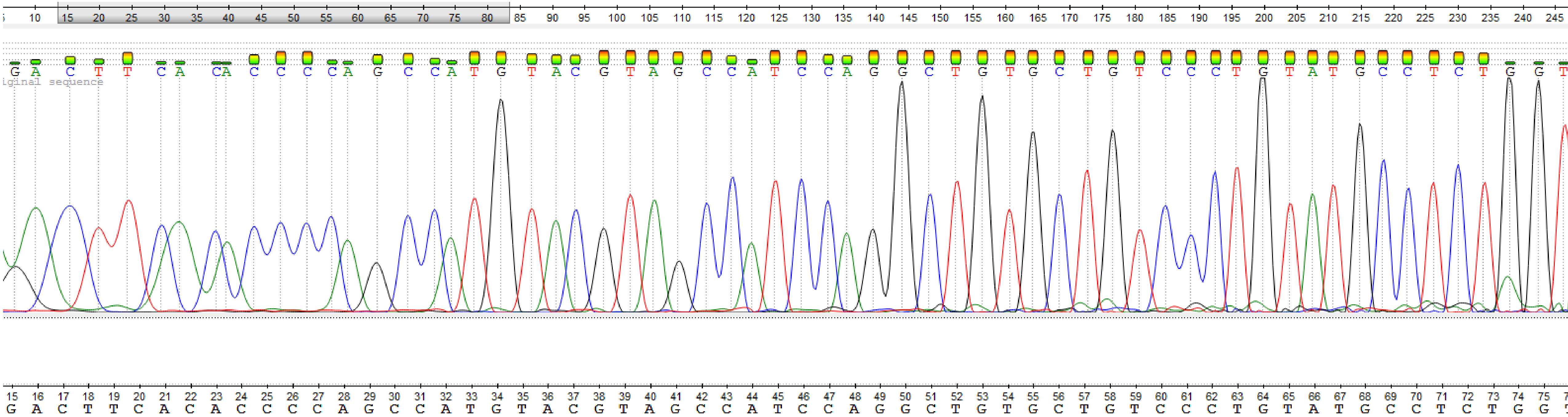

Download GenBank Graphics Sort by: E value

Mus musculus actin, beta (Actb), mRNA

Sequence ID: NM_007393.5 Length: 1935 Number of Matches: 2

Range 1: 484 to 547 GenBank Graphics Next Match Previous Match

Score	Expect	Identities	Gaps	Strand
119 bits(64)	3e-26	64/64(100%)	0/64(0%)	Plus/Plus

```
Query  1    GACCTTCAACACCCCAGCCATGTACGTAGCCATCCAGGCTGTGCTGTCCCTGTATGCCTC  60
            ||||||||||||||||||||||||||||||||||||||||||||||||||||||||||||
Sbjct  484  GACCTTCAACACCCCAGCCATGTACGTAGCCATCCAGGCTGTGCTGTCCCTGTATGCCTC  543

Query  61   TGGT  64
            ||||
Sbjct  544  TGGT  547
```

Download GenBank Graphics

Mus musculus leucine rich repeat containing 58 (Lrrc58), mRNA

Sequence ID: NM_177093.3 Length: 8592 Number of Matches: 1

Range 1: 2626 to 2683 GenBank Graphics Next Match Previous Match

Score	Expect	Identities	Gaps	Strand
91.6 bits(49)	6e-18	55/58(95%)	0/58(0%)	Plus/Plus

```
Query  7     CAACACCCCAGCCATGTACGTAGCCATCCAGGCTGTGCTGTCCCTGTATGCCTCTGGT  64
             ||| |||||||||||| |||||||||||||||||||||||||||||||||||||||||
Sbjct  2626  CAATACCCCAGCCATGTCTGTAGCCATCCAGGCTGTGCTGTCCCTGTATGCCTCTGGT  2683
```

Manuscript title: Validation of control genes and a standardised protocol for quantifying gene expression in the livers of *C57BL/6* and *ApoE-/-* mice

Authors: Priscilla EL Day[1], Karen F Chambers[1], Mark S Winterbone[1], Tatiana García-Blanco[1], David Vauzour[2], Paul A Kroon[1]
