# Supplementary material for: Validation of control genes and a standardised protocol for quantifying gene expression in the livers of C57BL/6 and ApoE−/− mice
Source: Sci Rep. 2018 May 24;8:8081. doi: 10.1038/s41598-018-26431-3 (PMC5967315; doi:10.1038/s41598-018-26431-3)
Supplement: Supplementary file 2 — Supplementary Figure 2 [file 41598_2018_26431_MOESM2_ESM.pdf]

Sequencing product and chromatogram from reverse primer (ACC AGA GGC ATA CAG GGA CA) -TGGCTGGGGTGTTGAAGGTCTCAAACATGATCTGGGTCATCTTTTCACGGTTGGCCTTAG

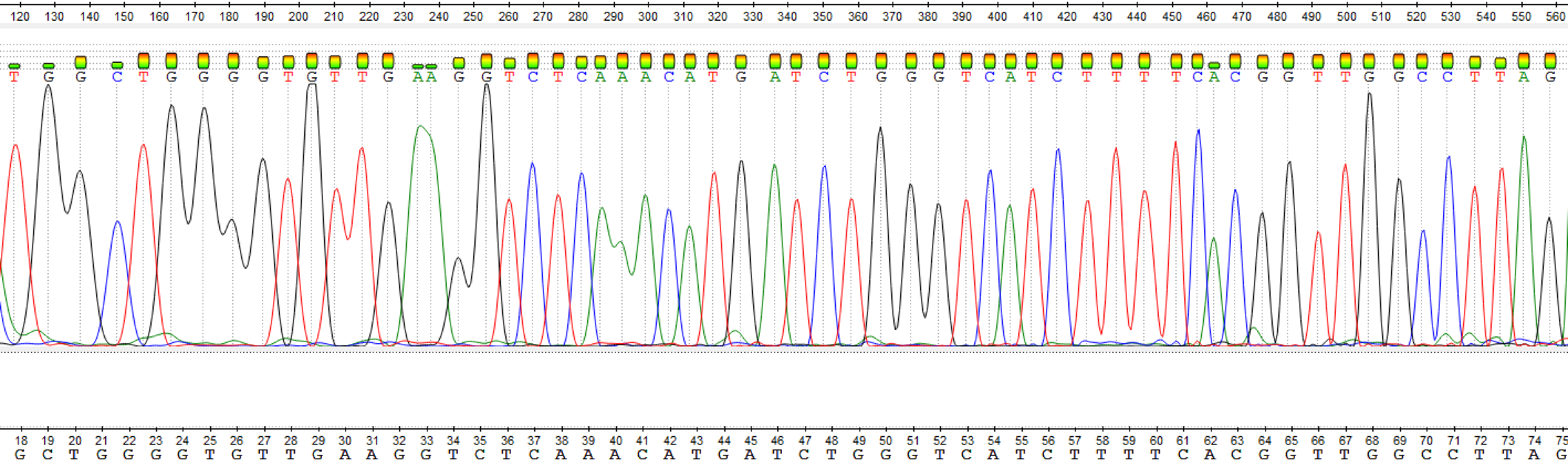

Download GenBank Graphics

Mus musculus actin, beta (Actb), mRNA

Sequence ID: NM_007393.5 **Length:** 1935 **Number of Matches:** 1

Range 1: 444 to 503 GenBank Graphics — Next Match Previous Match

Score	Expect	Identities	Gaps	Strand
111 bits(60)	4e-24	60/60(100%)	0/60(0%)	Plus/Minus

```
Query  1    TGGCTGGGGTGTTGAAGGTCTCAAACATGATCTGGGTCATCTTTTCACGGTTGGCCTTAG  60
            ||||||||||||||||||||||||||||||||||||||||||||||||||||||||||||
Sbjct  503  TGGCTGGGGTGTTGAAGGTCTCAAACATGATCTGGGTCATCTTTTCACGGTTGGCCTTAG  444
```

Download GenBank Graphics

Mus musculus leucine rich repeat containing 58 (Lrrc58), mRNA

Sequence ID: NM_177093.3 **Length:** 8592 **Number of Matches:** 1

Range 1: 2583 to 2639 GenBank Graphics — Next Match Previous Match

Score	Expect	Identities	Gaps	Strand
80.5 bits(43)	1e-14	55/60(92%)	3/60(5%)	Plus/Minus

```
Query  1     TGGCTGGGGTGTTGAAGGTCTCAAACATGATCTGGGTCATCTTTTCACGGTTGGCCTTAG  60
             ||||||||| |||   |||||||||||||||||||||||||||||||| ||||||||||
Sbjct  2639  TGGCTGGGGTATTG---GTCTCAAACATGATCTGGGTCATCTTTTCACTGTTGGCCTTAG  2583
```

Manuscript title: Validation of control genes and a standardised protocol for quantifying gene expression in the livers of *C57BL/6* and *ApoE-/-* mice

Authors: Priscilla EL Day[1], Karen F Chambers[1], Mark S Winterbone[1], Tatiana García-Blanco[1], David Vauzour[2], Paul A Kroon[1]
